# Manganese toxicity disrupts indole acetic acid homeostasis and suppresses the CO_2_ assimilation reaction in rice leaves

**DOI:** 10.1038/s41598-021-00370-y

**Published:** 2021-10-22

**Authors:** Daisuke Takagi, Keiki Ishiyama, Mao Suganami, Tomokazu Ushijima, Takeshi Fujii, Youshi Tazoe, Michio Kawasaki, Ko Noguchi, Amane Makino

**Affiliations:** 1 Faculty of Agriculture, Setsunan University, Hirakata, Osaka 573-0101 Japan; 2 Graduate School of Agricultural Science, Tohoku University, Sendai, Miyagi 980-8572 Japan; 3 Department of Applied Life Science, School of Life Sciences, Tokyo University of Pharmacy and Life Sciences, Hachioji, Tokyo 192-0392 Japan; 4 Present Address: Faculty of Agro-Food Science, Niigata Agro-Food University, Tainai, Niigata 959-2702 Japan; 5 Present Address: Faculty of Food and Agricultural Sciences, Fukushima University, Kanayagawa, Fukushima 960-1296 Japan

**Keywords:** Plant sciences, Photosynthesis, Plant breeding, Plant cell biology, Plant physiology

## Abstract

Despite the essentiality of Mn in terrestrial plants, its excessive accumulation in plant tissues can cause growth defects, known as Mn toxicity. Mn toxicity can be classified into apoplastic and symplastic types depending on its onset. Symplastic Mn toxicity is hypothesised to be more critical for growth defects. However, details of the relationship between growth defects and symplastic Mn toxicity remain elusive. In this study, we aimed to elucidate the molecular mechanisms underlying symplastic Mn toxicity in rice plants. We found that under excess Mn conditions, CO_2_ assimilation was inhibited by stomatal closure, and both carbon anabolic and catabolic activities were decreased. In addition to stomatal dysfunction, stomatal and leaf anatomical development were also altered by excess Mn accumulation. Furthermore, indole acetic acid (IAA) concentration was decreased, and auxin-responsive gene expression analyses showed IAA-deficient symptoms in leaves due to excess Mn accumulation. These results suggest that excessive Mn accumulation causes IAA deficiency, and low IAA concentrations suppress plant growth by suppressing stomatal opening and leaf anatomical development for efficient CO_2_ assimilation in leaves.

## Introduction

Mn is an essential nutrient for both terrestrial plants and animals^1^. With regard to terrestrial plants, Mn was first discovered in their ash, and McHargue^2^ proved that Mn is an essential nutrient. Mn possesses a wide variety of physiological functions in plant cells. For example, Mn activates more than 35 different enzymes such as chloroplast RNA-polymerase, and several enzymes involved in the tricarboxylic acid (TCA) cycle and shikimic acid pathway^3^. Mn is also directly involved in the physiological function of Mn-superoxide dismutase (SOD) as a cofactor to detoxify reactive oxygen species (ROS) and oxalate oxidase^3^. Among these physiological functions, the Mn cluster within the oxygen-evolving complex of photosystem II (PSII) is crucial in driving photosynthesis in terrestrial plants^4^.

Although Mn is indispensable for terrestrial plants, excess Mn accumulation in leaves causes Mn toxicity and reduces growth and crop yield^5,6^. The toxic effects of Mn are observed in various terrestrial plants, but the critical concentration for expressing toxicity varies depending on the plant species and genotype^5,7–9^. For example, *Zea mays* L. can show signs of Mn toxicity at an accumulation of 200 µg Mn g^−1^ (dry weight; D.W.) in the leaves^1^. In contrast, *Lupinus albus* L. or woody Mn hyperaccumulator species, such as *Gossia bidwillii*, can accumulate more than 10,000 µg Mn g^−1^ (D.W.) without Mn toxicity symptoms^10–12^. These differences in the critical concentration for Mn toxicity are derived from the different capacities of Mn compartments within cells^11^. Excessive Mn is sequestrated to vacuoles in a chelated form with organic acids (malate/citrate) to maintain the Mn concentration within cells^11^. However, when the vacuole capacity for storing Mn reaches the upper limit, the Mn concentration in the symplastic and apoplastic regions increases ^8,13^. In fact, Mn-sensitive plants showed a smaller vacuole capacity for retaining Mn than Mn-tolerant plants^7,11^. This interpretation has been validated by loss-of-function and overexpression mutants of tonoplast-localised Mn transporter proteins such as metal tolerant protein (MTP) 8 and calcium exchanger (CAX) 2, which showed a susceptible and tolerant phenotype to Mn toxicity, respectively^14–17^. In addition to these mechanisms, Mn-tolerant plants, such as *Helianthus annuus* L., use trichomes as Mn storage tissue to avoid an excessive increase in Mn concentration within cells^10,11^. However, this mechanism depends on the plant species and is not a generalised strategy to prevent Mn toxicity^11^.

Mn toxicity can be divided into two categories depending on the cell part of its onset: apoplastic and symplastic^18^. The primary symptom of apoplastic Mn toxicity in terrestrial plants is the appearance of brown spots containing oxidised phenolics, oxidised Mn [Mn^3+^ and Mn^4+^], and callose in the leaves^7,8,11,19–23^. Mn accumulation in the apoplast stimulates the expression of class III peroxidases (PODs), which undertake both H_2_O_2_ production through NADH-peroxidase activity and H_2_O_2_ consumption through guaiacol-peroxidase activity^13,22,23^. Furthermore, the concentrations of phenolics, which suppress NADH-peroxidase activity, are lowered in the apoplast and, as a consequence, H_2_O_2_ production is accelerated in the apoplast^13,24^. The H_2_O_2_ consumption reaction by the guaiacol activity of PODs also proceeds by utilising phenolics in the apoplast; subsequently, the intermediate phenolic oxidation product, phenoxyl radicals (PhȮ), oxidises Mn^2+^ to Mn^3+^^25^. Because PhȮ is regenerated to phenolics after the reaction with Mn^2+^, the continuous reactions of H_2_O_2_-production/consumption by PODs accumulate oxidised phenolics and oxidise Mn in the apoplast^19^.

Although brownish spots are an indicator of Mn toxicity in terrestrial plants, attenuation of plant growth by Mn toxicity is not always accompanied by the expression of brown spots in leaves^8,10,11,16,21,26,27^. Under excess Mn accumulation in the symplast, leaves show lower photosynthetic activity and lower chlorophyll (Chl) content^28–32^. Houtz et al.^28^ demonstrated a decrease in photosynthetic activity despite the absence of brown necrotic spots on leaves grown under high Mn concentrations, implying that a decrease in photosynthetic activity occurred independently of apoplastic Mn toxicity. This conditions is termed symplastic Mn toxicity^18^. The decreases in Chl concentration and photosynthetic activities have been hypothesised to be caused by photoinhibition by ROS; the decrease in photosystem I (PSI) content; the disturbance of Chl synthesis due to the inhibition of Fe absorption; Mn-binding to Rubisco instead of Mg; or stomatal dysfunction^33–38^. However, these hypotheses are often independently discussed at both in vivo and in vitro levels; therefore, the overall mechanisms by which symplastic Mn toxicity suppresses photosynthesis are less evident*.* Elucidating Mn toxicity, including both its apoplastic and symplastic mechanisms, would contribute to establishing a strategy for protecting terrestrial plants against excess Mn accumulation^32^. Currently, the strategy for preventing Mn toxicity depends on two mechanisms; limiting Mn absorption and transport or Mn sequestration from the cytosol to the vacuole^6^. These strategies are important in preventing Mn toxicity, but identifying the expression mechanisms of symplastic Mn toxicity would also be informative in manipulating Mn toxicity in terrestrial plants without limiting the Mn transportation system.

In this study, we investigated the mechanisms underlying symplastic Mn toxicity in suppressing photosynthetic activity in rice leaves. Under excess Mn conditions, rice also sequesters excess Mn to vacuoles through the tonoplast-localized Mn transporter^16^, or to the apoplast region in a *trans*-Golgi network-dependent manner^27^. Owing to these protective mechanisms, rice shows high tolerance toward Mn toxicity, which makes it easy to investigate the effect of excess Mn concentrations without severe necrosis and chlorosis^39^. Additionally, complete genetic information is available to investigate gene expression. Previous studies have indicated that photosynthetic electron transport activities on the thylakoid membrane and Rubisco isolated from leaves grown under high Mn concentrations were robust, although CO_2_ assimilation was substantially suppressed in leaves^28,35,40^. Based on these observations, we hypothesised that chloroplast proteins are not the primary targets of Mn toxicity, but that the factors that modulate CO_2_ assimilation are targeted under Mn toxicity. Here, we discuss how photosynthetic activity is limited under Mn-toxicity conditions from a wide range of physiological responses in plant cells.

## Results

### Phenotypes of rice grown under Mn-toxic conditions

**Figure 1:**
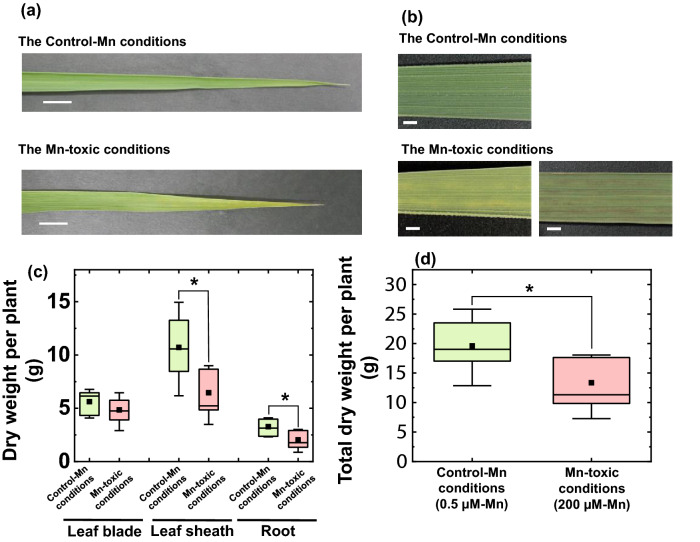
Phenotypes of rice plants grown under control and Mn-toxic conditions. The pictures of fully expanded leaf blades grown under control and Mn-toxic conditions are shown in (**a**), and (**b**) shows an enlarged picture of these. White bars indicate lengths of 1 cm (**a**) and 1 mm (**b**), respectively. (**c**) Dried weights of leaf blade, leaf sheath, and root, respectively. (**d**) The sum of the dried weights is shown in (**c**). Data are shown as box plots obtained by independent biological replicates (n = 9), black squares indicate the mean value, and bars indicate the range of the maximum or minimum data within a 1.5 × interquartile range (IQR). The green boxes indicate the results of the control conditions, and the red boxes indicate those of the Mn-toxic conditions. Asterisks show significant differences between the control and Mn-toxic conditions (*p < 0.05, Kruskal–Wallis test).

To study the effects of Mn toxicity, *Oryza sativa* L. ‘Nipponbare’ was grown in hydroponic culture under high Mn concentrations (200 µM; Mn-toxic conditions) to evaluate the effect of Mn accumulation on leaf physiological responses without severe chlorosis and necrosis in rice leaves during their growth^27^. The following studies were conducted on fully expanded leaves for 70 days after germination (see Materials and Methods). Under Mn-toxic conditions, the leaves showed pale green and brown-coloured sections, especially at the tips as observed previously (Fig. 1a,b)^16,41^. Compared with the control conditions, Mn-toxic conditions decreased the total dry weight of rice, especially the dry matter of the leaf sheath and root (Fig. 1c,d).

**Figure 2:**
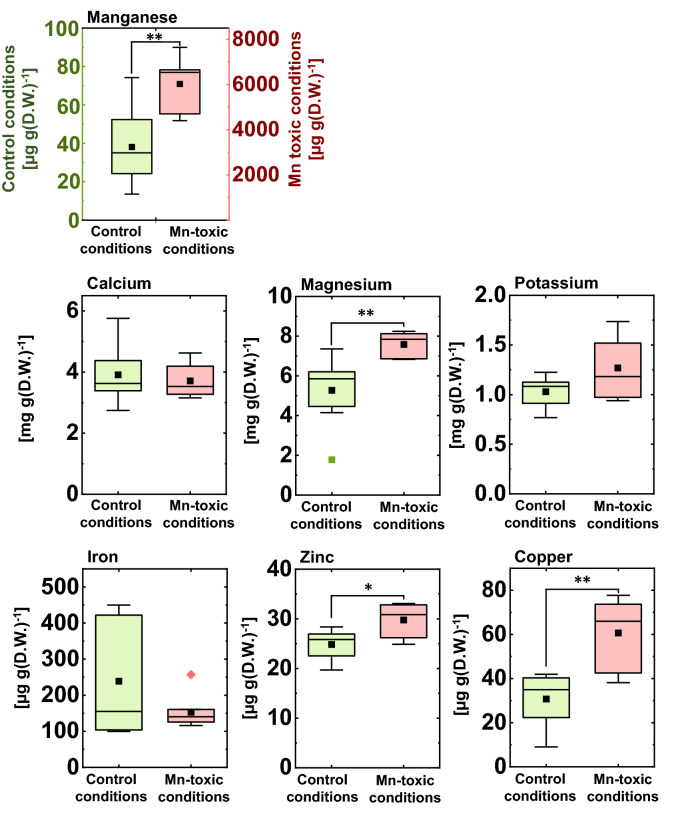
Mineral concentration in the rice leaf blade. Data are shown as box plots obtained by independent biological replicates (control conditions n = 8, Mn-toxic conditions n = 7), black squares indicate the mean value, and bars indicate the range of the maximum or minimum data within a 1.5 × interquartile range (IQR). Green boxes indicate the results of the control conditions, and red boxes indicate those of the Mn-toxic conditions. Asterisks indicate significant differences between the control and Mn-toxic conditions (*p < 0.05, **p < 0.01, Kruskal–Wallis test).

To study the effects of high Mn application on leaf mineral composition, leaf mineral concentrations were quantified. The leaf Mn concentration was significantly increased under Mn-toxic conditions (Fig. 2). This concentration was comparable to that of rice plants, which exhibited growth inhibition when grown at a concentration of 1000 µM Mn for 3 weeks^42^. The K and Ca concentrations were similar under the control and Mn-toxic conditions (Fig. 2). The Fe concentration did not show a significant difference between the control and Mn-toxic conditions; however, leaf Fe concentrations under Mn-toxic conditions were distributed to a lower level compared to those under the control conditions (Fig. 2). In contrast, the Mg, Zn, and Cu concentrations increased under Mn-toxic conditions (Fig. 2). These results demonstrated decreased growth and Mn toxicity symptoms accompanied by Mn accumulation in leaves grown under Mn-toxic conditions.

### Mn-toxic conditions suppressed CO_2_ assimilation by limiting stomatal conductance in leaves

**Figure 3:**
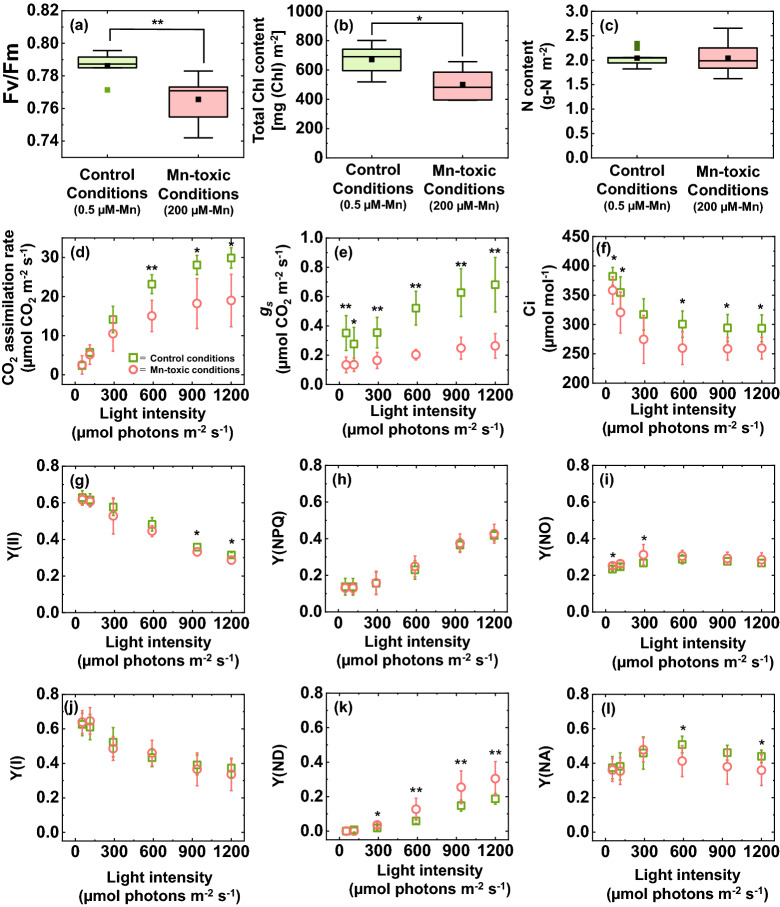
Photosynthetic activities in leaves grown under control and Mn-toxic conditions. The maximum quantum yield of PSII (Fv/Fm) is shown in (**a**). (**b**,**c**) Show the total Chl and N concentrations in the leaves, respectively. The CO_2_ assimilation rate (**d**), stomatal conductance (*g*_*s*_) (**e**), and intercellular CO_2_ concentration (**f**) are shown. For the PSII photosynthetic parameters, the quantum yields of PSII [Y(II)] (**g**), non-photochemical quenching [Y(NPQ)] (**h**), and non-radiative decay [Y(NO)] (**i**) are shown. For PSI photosynthetic parameters, the quantum yields of PSI [Y(I)] (**j**), non-photochemical quenching at the donor side [Y(ND)] (**k**), and non-photochemical quenching at the acceptor side [Y(NA)] (**l**) are shown. Data of photosynthetic parameters are shown as means with standard deviation (SD) (n = 7). The results for Fv/Fm (n = 7) and total Chl (n = 7) and N (n = 9) concentrations are shown as box plots. All data were obtained from independent biological replicates according to the indicated numbers. Black squares indicate the mean value, and bars indicate the range of the maximum or minimum data within a 1.5 × interquartile range (IQR). Asterisks show significant differences between the control and Mn-toxic conditions (*p < 0.05, **p < 0.01, Kruskal–Wallis test).

The maximum quantum yield of PSII (Fv/Fm) was significantly but marginally decreased under Mn-toxic conditions (Fig. 3a). The total Chl content in the leaves also decreased under Mn-toxic conditions (Fig. 3b). These results indicated that Mn toxicity causes PSII photoinhibition. In contrast, the total N concentration did not significantly differ between the control and Mn-toxic conditions (Fig. 3c). Based on the linear function between the N and Rubisco concentrations in the leaves^43^, this result indicated that the quantity of Rubisco was less affected by Mn toxicity.

Next, the steady-state photosynthetic activities were measured under ambient air conditions (40 Pa CO_2_ and 21 kPa O_2_). The CO_2_ assimilation rate was significantly decreased by approximately 39% under high light irradiance as a result of Mn-toxic treatments (Fig. 3d). The Mn-toxic treatments showed lower stomatal conductance (*g*_*s*_) than the control plants (Fig. 3e). These stomatal responses to the increase in Mn concentration were consistent with previous studies^28,30,34,44,45^. The internal CO_2_ concentration in leaves (Ci) was lower under Mn-toxic conditions than under control conditions (Fig. 3f).

In addition to the CO_2_ assimilation rate, photosynthetic electron transport activities in both PSII and PSI were analysed. The quantum yield of PSII [Y(II)] was decreased by approximately 9% under Mn-toxic conditions than under control conditions at high light irradiance (Fig. 3g). This result indicated that the electron transport activity in PSII was suppressed at high light irradiance, but compared with the CO_2_ assimilation rate, the change in Y(II) was marginal. The quantum yield of non-photochemical quenching [Y(NPQ)] increased with increasing light intensity under both control and Mn-toxic conditions (Fig. 3h). The quantum yield of non-radiative energy loss [Y(NO)] differed between the control and Mn-toxic conditions under low light irradiance; however, this difference was masked under high light irradiance (Fig. 3i). These results showed that the photoprotective mechanisms in PSII are robust, but the redox state in PSII can be perturbed under Mn toxicity. The quantum yield of PSI [Y(I)] showed similar kinetics to that of Y(II), and no significant differences were observed between the control and Mn-toxic conditions (Fig. 3j). The quantum yield of non-photochemical quenching at the donor side of PSI [Y(ND)] was higher under Mn-toxic conditions than under control conditions at high light irradiance (Fig. 3k). In contrast, the quantum yield of non-photochemical quenching at the acceptor side of PSI [Y(NA)] was lower under Mn-toxic conditions than under control conditions at high light irradiance (Fig. 3l). These results indicated that the whole-chain photosynthetic electron transport rate was less affected under Mn-toxic conditions; however, the photosynthetic electron transport reaction was limited at the donor side of PSI, and PSI was more oxidised under Mn-toxic conditions than under the control conditions.

Previous studies have suggested that Mn-binding to Rubisco causes a decrease in photosynthesis under Mn toxicity^31,35^. In fact, Mn does bind Rubisco, and an in vitro study indicated that the substitution of Mg^2+^ with Mn^2+^ decreases the specificity of Rubisco for CO_2_^46,47^. However, the contribution of Mn-binding Rubisco to decrease CO_2_ assimilation reactions remains unknown under Mn-toxic conditions in vivo^35,40^. To address this question, we simulated the change in CO_2_ assimilation rate in rice leaves containing Mg^2+^- or Mn^2+^-binding Rubisco using a photosynthetic biochemical model^48^. In this simulation, leaf Rubisco content was assumed to be 5.3 µmol m^−2^ in leaves based on a previous study in both cases^49^, and the enzymatic parameters of Mg^2+^- and Mn^2+^-binding Rubisco were employed by Makino et al.^50^ and Bloom and Kameritsch^47^, respectively (see Materials and Methods). The calculated CO_2_ assimilation rate using Mg^2+^-binding Rubisco kinetics showed well-established kinetics and its Γ* value was 3.1 Pa of chloroplastic partial pressure of CO_2_ (Cc) (Supplementary Fig. S1a). In contrast, the calculated CO_2_ assimilation rate using Mn^2+^-binding Rubisco kinetics was negative below Cc = 50 Pa because Γ* value was 45.6 Pa of Cc, and it greatly decreased compared to that using Mg^2+^-binding Rubisco kinetics (Supplementary Fig. S1a). In addition to the CO_2_ assimilation rate, the photorespiration rate was simulated. Compared to Mg^2+^-binding Rubisco, Mn^2+^-binding Rubisco showed a higher photorespiration rate (Supplementary Fig. S1b). However, the difference between the photorespiration rates of Mg^2+^- and Mn^2+^-binding Rubisco only reached approximately 5 µmol CO_2_ m^−2^ s^−1^ (Supplementary Fig. S1b). These results indicate that the increase in photorespiration rate in Mn^2+^-binding Rubisco cannot compensate for the difference in the CO_2_ assimilation rate of Mg^2+^-binding Rubisco. That is, the binding of Mn^2+^ to Rubisco instead of Mg^2+^ should inhibit the photosynthetic electron transport activity entirely, but such a drastic decrease in photosynthetic electron transport activity was not observed (Fig. 3g,j). Moreover, the relationship between *g*_s_ and the CO_2_ assimilation rate did not show any decrease in the CO_2_ assimilation rate independent of the change in *g*_*s*_ (Supplementary Fig. S2)*.* These results indicated that, rather than the change in the enzymatic properties of Rubisco, the stomata limited CO_2_ diffusion into the leaves suppressed the CO_2_ assimilation rate.

### Carbohydrate metabolism in leaves grown under Mn-toxic conditions

**Figure 4:**
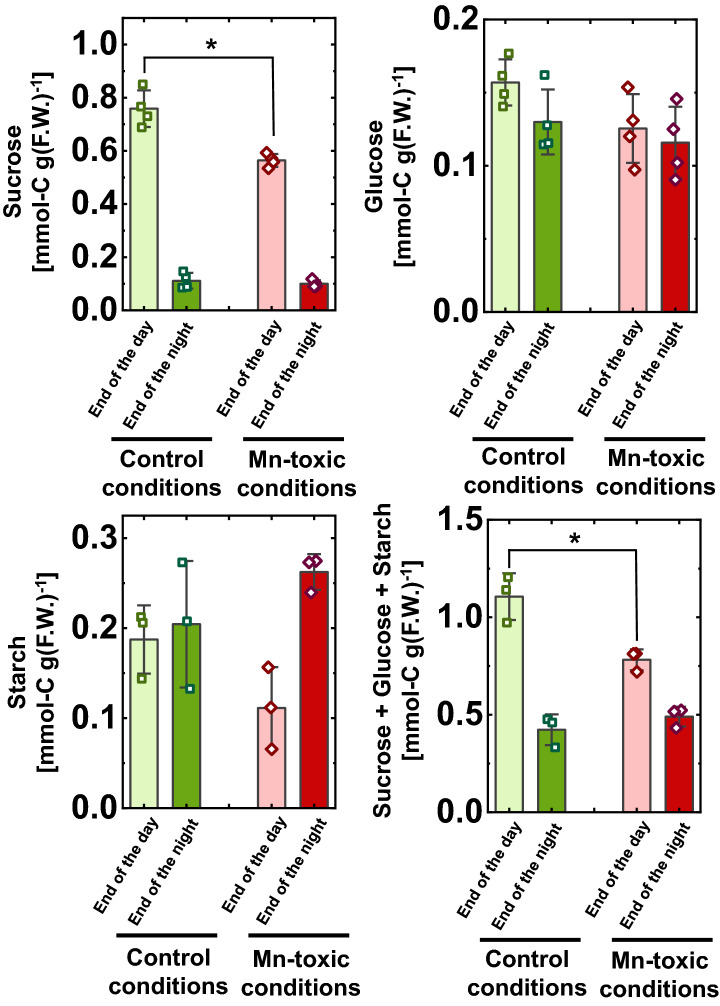
Carbohydrate content in leaf blades at the end of the day and end of the night. Data are shown as means with standard deviation (SD) obtained from independent biological replicates (n = 3–4), and squares and diamonds show the distribution of the raw data. Asterisks show significant differences between the control and Mn-toxic conditions (*p < 0.05, Kruskal–Wallis test).

To examine whether the suppression of the CO_2_ assimilation rate by excessive Mn accumulation affects carbon acquisition during growth, the major carbohydrate concentrations were quantified in the leaves. Leaves were sampled at both the end of the day and at the end of the night to visualise the CO_2_ assimilation activity during day^51^. At the end of the day, Mn toxicity treatments lowered the sucrose concentrations in the leaves compared to the control plants (Fig. 4). In contrast, at the end of the night, the sucrose concentration was similar under both control and Mn-toxic conditions (Fig. 4). Significant differences in glucose and starch concentrations were not detected between the growth conditions at the end of the day and at the end of the night (Fig. 4). The sum of the sucrose, glucose, and starch concentrations was lower in leaves grown under Mn-toxic conditions at the end of the day, but was similar with the growth conditions at the end of the night (Fig. 4). These results indicate that carbon acquisition is suppressed, corresponding to lower CO_2_ assimilation activities, in leaves grown under Mn-toxic conditions.

### Effects of Mn toxicity on mitochondrial respiration in the leaves

**Figure 5:**
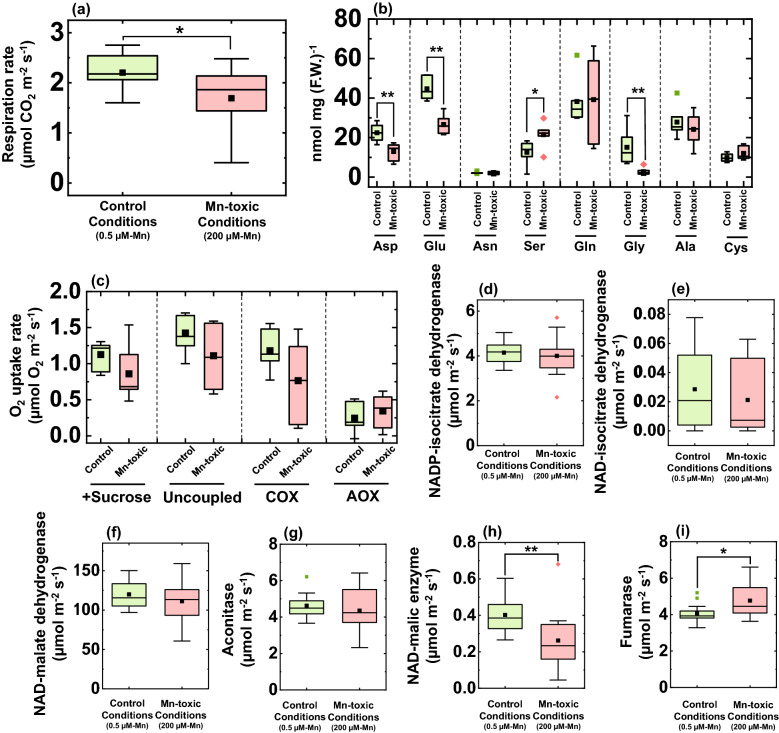
Mitochondrial respiration activities and amino acid contents in leaf blades grown under control and Mn-toxic conditions. (**a**) Leaf respiration rate (n = 12–13) in the dark and (**b**) sucrose feeding respiration activities and mitochondrial respiratory electron transport activities depending on ATP synthase (uncoupled), COX, and AOX (n = 7). (**c**) Amino acid content in leaves sampled in the middle of the night (n = 6). (**d**–**i**) Enzyme activities involved in the TCA cycle (n = 13–14). Data are shown as box plots, black squares indicate the mean value, and bars indicate the range of the maximum or minimum data within a 1.5 × interquartile range (IQR). All data were obtained from independent biological replicates according to the indicated numbers. The green boxes indicate the results under control conditions, and the red boxes indicate those under Mn-toxic conditions. Asterisks represent significant differences between the control and Mn-toxic conditions (*p < 0.05, **p < 0.01, Kruskal–Wallis test).

Carbohydrates fixed during the day are metabolised at night and consumed at a constant rate from the beginning of the night to the dawn^52^. Therefore, the difference in carbohydrate concentrations between the end of the day and the end of the night indicates the carbon catabolic activity during night^51^. To confirm whether carbon catabolism is suppressed under Mn-toxic conditions (Fig. 4), the leaf respiration rate was examined under control and Mn-toxic conditions. The respiratory CO_2_ emission rate decreased under Mn-toxic conditions (Fig. 5a). To examine the effect of the change in respiration activities on carbon catabolism, the leaf amino acid content was quantified during the night^53^. Subsequently, the Asp, Glu, and Gly contents were significantly decreased, and the Ser content increased under Mn-toxic conditions (Fig. 5b). These results indicate that carbon catabolism is affected by Mn-toxic conditions during the night.

Next, we investigated the effects of Mn toxicity on the mitochondrial enzymes involved in respiration. First, the respiratory electron transport activities were measured in vivo in the presence of sucrose and mitochondrial electron transport inhibitors using an aqueous phase O_2_-electrode^54^. In the absence of inhibitors, the O_2_ consumption rate did not differ between the control and Mn-toxic conditions (Fig. 5c). CCCP dissipated mitochondrial membrane potential (Δψ_m_) and similarly stimulated O_2_ consumption rate in both leaves grown under the control and Mn-toxic conditions (Fig. 5c). The COX and AOX activities in the presence of CCCP and sucrose were determined by adding n-propyl gallate and KCN. The control and Mn-toxic conditions showed similar O_2_ consumption rates depending on the COX and AOX activities (Fig. 5c). The effect of Mn toxicity on mitochondrial respiratory electron transport activity was reproduced when these activities were determined on a leaf fresh weight basis (Supplementary Fig. S3). These results indicated that under sucrose feeding, the mitochondrial respiratory electron transport activities were similar between the control and Mn-toxic conditions.

To understand the effects of Mn toxicity on the enzyme activities of the TCA cycle, the six types of TCA cycle enzyme activities were determined in terms of both leaf area (Fig. 5) and fresh-weight basis (Supplementary Fig. S4). NADP-isocitrate dehydrogenase, NAD-isocitrate dehydrogenase, NAD-malate dehydrogenase, and aconitase showed similar activities under control and Mn-toxic conditions (Fig. 5d–g, Supplementary Fig. S4). NAD-malic enzyme activity decreased under Mn-toxic conditions (Fig. 5h, Supplementary Fig. S4), whereas the fumarase activity increased (Fig. 5i, Supplementary Fig. S4). These results indicated that Mn toxicity did not completely suppress the enzyme activities of the TCA cycle, but specifically stimulated fumarase and suppressed NAD-malic enzyme activities.

### Difference in stomatal development in the leaves between the control and Mn-toxic conditions

**Figure 6:**
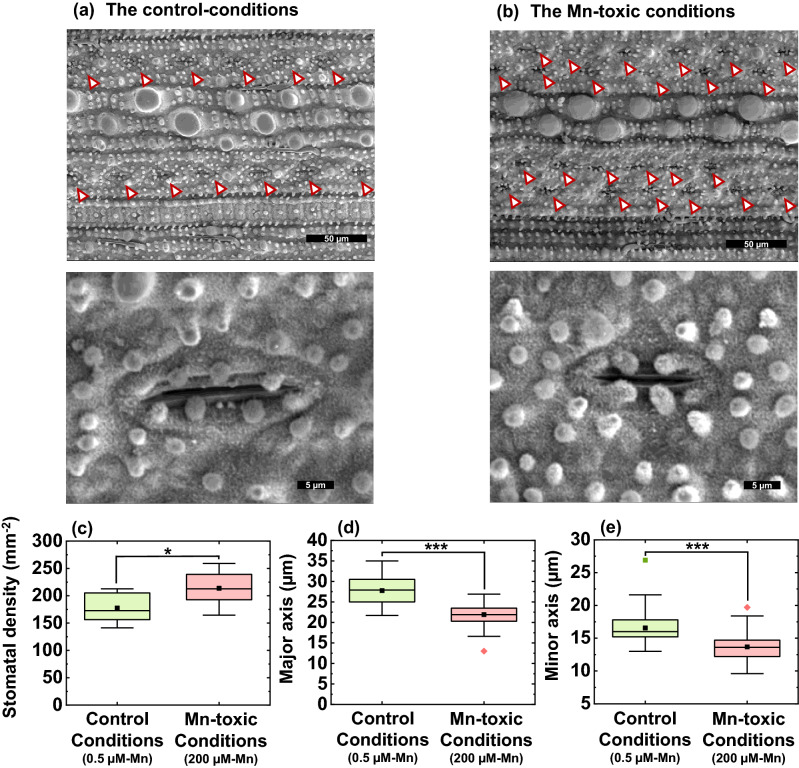
Stomatal structures and characteristics in the rice leaf blade. Pictures of the stomata on the abaxial side of the leaf blade grown under control (**a**) and Mn-toxic (**b**) conditions. The upper pictures focus on the stomatal distributions, and the lower pictures focus on the single stomata complex. (**c**) stomatal density (n = 10–12). (**d**,**e**) Show the major and the minor axis of the stomatal complex, respectively (n = 53–63). Data are shown as box plots, black squares indicate the mean value, and bars indicate the range of the maximum or minimum data within a 1.5 × interquartile range (IQR), and these data were obtained from the six independent biological replicates and independent technical replicates as indicated. The green boxes indicate the results under control conditions, and the red boxes indicate those under Mn-toxic conditions. Asterisks represent significant differences between the control and Mn-toxic conditions (*p < 0.05, ***p < 0.001, Kruskal–Wallis test).

To examine whether the decrease in *g*_*s*_ under Mn-toxic conditions was related to the change in stomatal structure and development, the stomatal complex on the abaxial side of a leaf was monitored. The stomatal complex, consisting of guard and subsidiary cells, was located linearly to the vein under control conditions (Fig. 6a). However, under Mn-toxic conditions, the stomatal complex showed a more scattered distribution (Fig. 6b). The stomatal density increased under Mn-toxic conditions (Fig. 6c). In contrast, the size of the stomatal complex evaluated by the maximum major and minor axes was smaller under Mn-toxic conditions than under control conditions (Fig. 6a,b,d,e). The changes in stomatal size were consistent with those of a previous study on rice^55^. These results indicate that Mn-toxic conditions change stomatal development and patterning in the leaves.

### Anatomical changes within a leaf blade grown under Mn-toxic conditions

**Figure 7:**
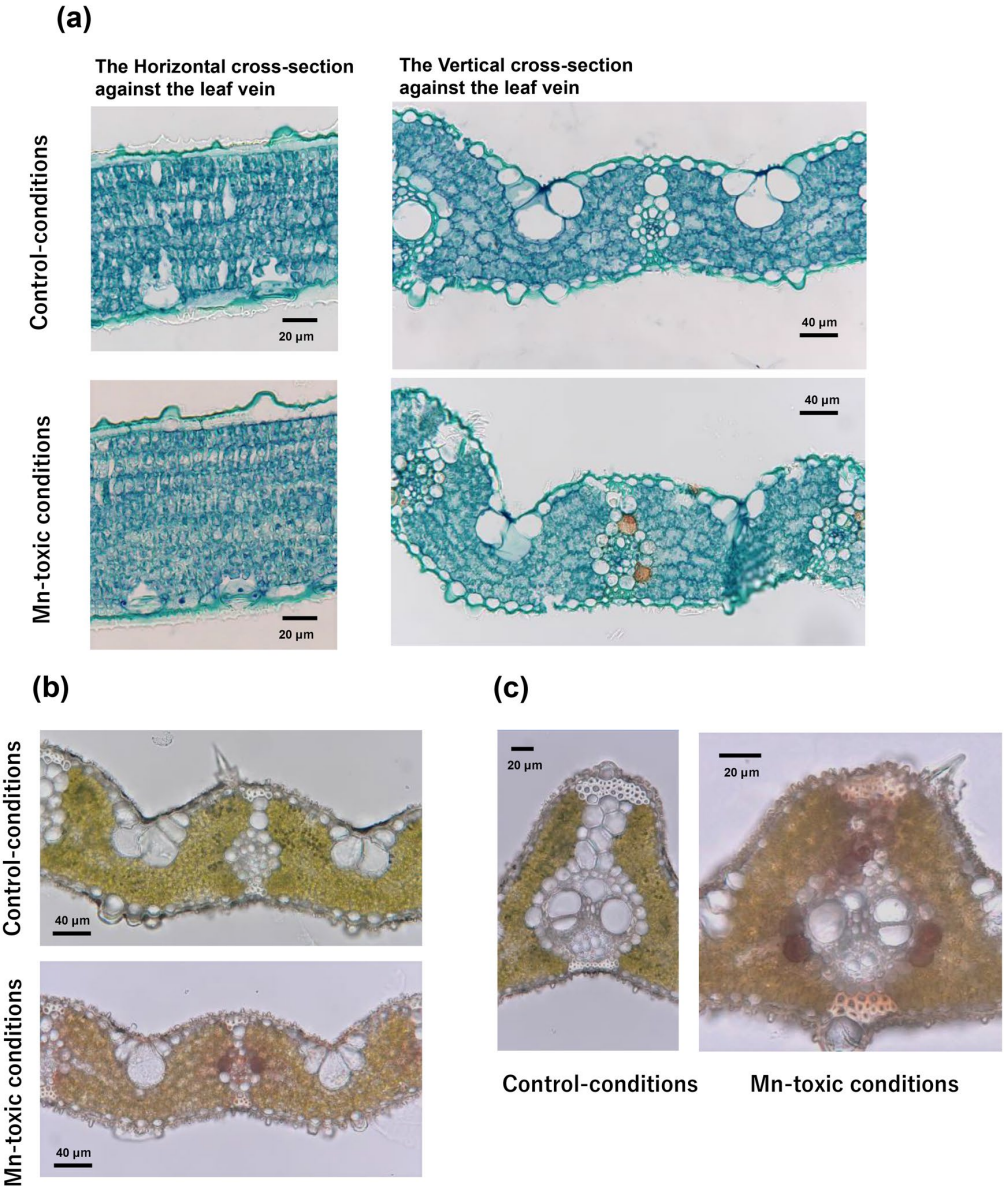
Characteristics of the leaf section grown under control and Mn-toxic conditions. (**a**) Fixed leaf sections sectioned from the horizontal and vertical side against the leaf vein. Fresh leaf sections of the small (**b**) and large (**c**) bundles. Black bars show the scales.

Following the change in stomatal development on the leaf epidermis, the leaf anatomy was also examined. Figure 7a shows the horizontal and vertical cross-sections of the leaves against the vein. Apparent internal air spaces were observed within leaves grown under control conditions. In contrast, cells were highly condensed in leaves grown under Mn-toxic conditions, and the internal air spaces were hardly observed (Fig. 7a). These results showed that, in addition to the stomatal complex, leaf anatomical development was altered under Mn-toxic conditions.

The vertical cross-section revealed that the bundle-sheath cells were brownish under Mn-toxic conditions (Fig. 7a). To investigate whether the brown spots were artifacts caused by the chemical fixing treatment, intact leaf sections were also studied. Subsequently, we found that brownish spots natively existed in the bundle sheath cells, in both small and large bundles grown under Mn-toxic conditions (Fig. 7b,c). These results indicated that under Mn-toxic conditions, rice leaves caused apoplastic Mn toxicity-like symptoms in addition to symplastic Mn toxicity, and the symptoms were more evident in the bundle-sheath cells in rice leaves.

### Auxin content in the leaves and mRNA expression of auxin-responsive genes

**Figure 8:**
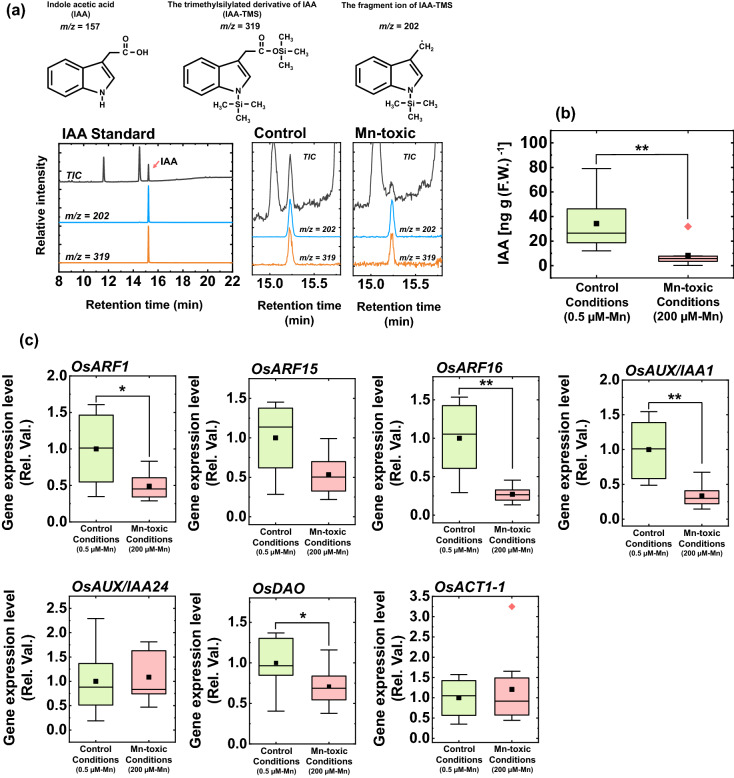
Leaf indole acetic acid (IAA) concentration and gene expression analysis relating to IAA and stomata development. (**a**) The result of GC–MS analysis targeting IAA, together with the chemical structures of IAA and its derivatives. The total ion chromatography (TIC) and chromatographs of *m*/*z* = 202 and *m*/*z* = 319 in the standard IAA solution and the leaf extract from plants under control and Mn-toxic conditions are shown. The retention time of the silylated IAA was 15.2 min. (**b**) The IAA concentrations in the leaf blade (n = 8). (**c**) The results of gene expression analysis involving IAA-responsive gene and stomatal patterning (n = 8). All data were obtained from independent biological replicates according to the indicated numbers. The quantified results were normalized to *OsATC1-2* expression basis. Asterisks show significant differences between the control and Mn-toxic conditions (*p < 0.05, **p < 0.01, Kruskal–Wallis test).

Our findings revealed that excess Mn accumulation affects the developmental process of leaves. IAA is an important hormone determining cell fate and differentiation in plants^56^. Morgan et al.^57^ reported that excess Mn application to cotton plants increased IAA oxidation activity in the leaf extract. This observation implies that the auxin concentration could be lowered by accelerated auxin degradation. To examine this possibility, the IAA concentration in the leaves was quantified using GC–MS. Figure 8a shows the total ion chromatograms (TIC) with fragment ion chromatograms of the 202 (*m*/*z*) and 319 (*m*/*z*) of the IAA standard and leaf extract from the control and Mn-toxic conditions (Fig. 8a).This analysis revealed that the IAA concentration was significantly decreased by 76% in leaves under Mn-toxic conditions (Fig. 8b).

To confirm that the decrease in IAA concentration affects physiological reactions within the leaves, IAA-responsive gene expression was investigated. *OsARF1*, *OsARF15*, *OsARF16*, *OsAUX*/*IAA1,* and *OsAUX*/*IAA24* showed increased expression levels with an increase in IAA concentration in the leaves^58–60^. Among these genes, the expression levels of *OsARF1*, *OsARF16,* and *OsAUX*/*IAA1* were significantly decreased under Mn-toxic conditions (Fig. 8c). *OsARF15* showed similar trends to other *ARF* genes, but the difference was not significant between the growth conditions. In contrast, *OsAUX*/*IAA24* and *OsACT1-1* showed comparable expression levels between the control and Mn-toxic conditions (Fig. 8c). The results of the auxin-responsive gene expression indicated a lower IAA concentration in leaves under Mn-toxic conditions, in agreement with the results of GC–MS, although all genes did not respond equally.

To regulate auxin concentration in plant cells, DIOXYGENASE FOR AUXIN OXIDATION (DAO) undertakes IAA catabolism to maintain IAA homeostasis by oxidising IAA^61^. Because Mn-toxicity stimulates oxidative IAA degradation in vitro^57^, we hypothesised that *OsDAO* is upregulated following an increase in Mn concentration; however, the transcriptional activation of *OsDAO* was suppressed under Mn-toxic conditions (Fig. 8c). *OsDAO* expression is activated under high IAA concentrations to maintain IAA concentrations in plant tissues^61^. Based on this homeostatic response of *OsDAO*, the decrease in *OsDAO* expression under Mn-toxic conditions might be a consequence of the lower IAA concentration in leaves to avoid further inactivation of IAA.

## Discussion

In acidic or waterlogged agricultural fields, Mn is easily released from the soil and causes Mn toxicity in crop plants^18^. In addition to current agricultural situations, the effects of climate change, such as the rise in the atmospheric temperature and the frequency of flooding or acidic rainfall, can further accelerate Mn dissolution in soils^32^. Therefore, the mechanisms of Mn toxicity should be elucidated to manipulate the future threat of increasing Mn availability in soils including agricultural lands. In this study, we aimed to investigate the mechanisms of symplastic Mn toxicity in rice. We propose that the disturbance of IAA homeostasis is one of the critical causes of symplastic Mn toxicity in rice leaves.

We suggest that the phenotype of the symplastic Mn toxicity is a result of IAA deficiency in the leaves. Here, we directly demonstrated that the leaf IAA concentration decreased under Mn toxicity along with the changes in *ARF* and *AUX*/*IAA* gene expression levels (Fig. 8).Similar to the results for rice, common bean leaves showed a change in the expression of microRNAs which target ARF and the auxin-signal related F-box protein (TIR1) under Mn toxicity^62^. Among other essential heavy metal elements, the specific effects of their excess accumulations on the IAA degradation activity have not been reported in terrestrial plants^3^. Therefore, we suggest that Mn toxicity specifically affects the auxin signalling pathway by stimulating IAA degradation in various terrestrial plants. When IAA deficiency is caused by the overexpression of GRETCHEN HAGEN3 which conjugates IAA to amino acids or the anion peroxidase which oxidatively inactivates IAA, the leaf cell size becomes smaller and the leaf anatomy shows more condensed cell structures^63–65^. Alternatively, transgenic plants that inhibit auxin signalling or auxin polar transport activities can represent IAA-deficient phenotype^66–69^. Moreover, stomatal development is also regulated by auxin signalling^70^. Exogenous IAA application decreased stomatal density in a concentration-dependent manner; whereas the attenuation of ARF-dependent auxin signal transduction increased it^71,72^. Based on these developmental responses, Mn toxicity causes IAA deficiency in leaves and disturbs optimal leaf and stomatal development by altering the auxin-signalling cascade involving *ARF* genes. In addition to the contribution of IAA to leaf development, IAA activates H^+^-ATPase embedded in the plasma membrane of guard cells and stimulates stomatal opening^73,74^. Generally, the terrestrial plants with smaller stomata and higher stomata density show higher transpiration ability than those with larger stomata and lower density^75^. Therefore, the decrease in IAA concentration under Mn toxicity would diminish the structural advantage of efficient transpiration (Figs. 3e and 6). However, the regulation of stomatal opening is quite complex, and the ROS and organic acid composition are also act as determinants of stomatal movement^74^. Considering the stimulation of oxidative stress under Mn toxicity (see the following discussion), the decrease in IAA might not be the only cause of stomatal dysfunction. Interestingly, fumarase activity increased under Mn toxicity (Fig. 5i). Because the increase in malate/fumarate ratio is important for stomatal opening^76^, the increased fumarase activity might be a counteraction to stomatal dysfunction under Mn toxicity. Currently, we cannot determine the molecular mechanism underlying the decrease in IAA concentrations under Mn toxicity. Further research is required to elucidate the factors that cause IAA deficiency. Because IAA is transported through the apoplast, the increase in POD activity in the apoplast might influence IAA catabolism under Mn toxicity^63,64,77,78^. Alternatively, there is a possibility that the antagonistic effects of excess Mn on other metals in an unknown molecular target may increase IAA degradation activity under excess Mn accumulation in rice leaves^32^.

We suggest that the prolonged limitation of the CO_2_ assimilation reaction and stimulation of photorespiration by stomatal closure caused oxidative stress by ROS under Mn toxicity. Even when the CO_2_ availability within chloroplasts is limited by stomatal closure, O_2_ can diffuse within the leaves; therefore, Rubisco drives photorespiration and maintains Y(II)^79^. When photorespiration is stimulated, the thylakoid lumen is more acidified owing to the lower ATP consumption rate in the photorespiration reaction compared with that in the carboxylation reactions in the Calvin-Benson cycle^80,81^. Furthermore, the photosynthetic electron transport reaction is limited at cytochrome *b*_*6*_*f* due to the lowering of luminal pH, which increases Y(ND)^80,81^. These photosynthetic characteristics were consistent with the results for Mn toxicity (Fig. 3). That is, our results suggest that Mn toxicity stimulates photorespiration by limiting stomatal opening. The observed increase in photorespiration activity can also be supported by the change in the Gly/Ser ratio under Mn-toxic conditions (Fig. 5b). A previous study reported that the limitation of the CO_2_ assimilation rate by drought stress with a decrease in *g*_*s*_ stimulates PSI photoinhibition rather than PSII photoinhibition by ROS^82^. A similar situation would occur under Mn toxicity, which is why the decrease in PSII activity is modest, although PSI inhibition is accentuated under Mn-toxic conditions^31,37,44^. The stimulation of oxidative stress is supported by the upregulation of ROS-scavenging enzyme activities under Mn toxicity in various terrestrial plants^38,45^, and indeed we observed a decrease in PSI reaction centre chlorophyll, P700, content in leaves under Mn toxicity (Supplementary Fig. S5). Previous studies have proposed that Mn toxicity decreases PSI content by attenuating Fe absorption^83^. Mn and Fe share some transporters for their uptake in rice roots such as the Natural Resistance-Associated Macrophage Protein (NRAMP)^84^. OsNRAMP5 is a major Mn transporter regulating the inward flow of Mn from soil to exodermal cells in concert with MTP9 which carries the outward flow of Mn from exodermal to endodermal cells^85–87^. Chen et al.^16^ and Tsunemitsu et al.^88^ suggested that excess Mn accumulation in the cytosol may downregulate Mn uptake activity by OsNRAMP5 through unrevealed post-transcriptional regulation. In other words, the lower distribution of Fe concentration might be caused by post-transcriptional regulation of the Mn uptake system. However, the suppression of Fe uptake by the OsNRAMP5 regulatory system would be minor because the rice *osnramp5* mutant did not show a significant decrease in Fe concentration in leaves^16,89^. This is because Fe uptake is compensated by another Fe transporter such as Yellow Stripe-Like (YSL)15, which is induced by excess Mn toxicity conditions^90^. Thus, we could not detect any significant differences in Fe concentrations between the control and Mn-toxic conditions (Fig. 2). Interestingly, YSL15 expression levels are also increased under Fe deficiency, in addition to excess Mn conditions^89,90^. From this observation, we suggest that rice detects some changes in Fe concentration in whole plants caused by excess Mn accumulation. However, Fe concentration was maintained within a sufficient range for Fe nutrition in rice in this study [from 70 to 300 µg g^−1^ (D.W.)]^91^. Therefore, the primary cause of the decrease in PSI content was not Fe deficiency. It is worth noting that inhibition of PSI synthesis caused an increase in Y(NA), instead of Y(ND) during steady-state photosynthesis^92^. Hence, we suggest that the decrease in PSI content due to Mn toxicity is caused by PSI photoinhibition, as a consequence of limited CO_2_ assimilation. In addition to this possibility for the lower distribution of Fe concentration under Mn-toxic conditions, we found that PSI concentration was significantly decreased under Mn toxicity conditions (Supplementary Fig. S5). PSI is a major Fe-containing supercomplex in the thylakoid membrane^3^. That is, the decrease in PSI content could also modulate Fe concentration in leaves.

Other nutrients such as Ca, Mg, and Zn were also reported to be deficient in Mn toxicity^3^. We did not observe a decrease in these minerals in the leaves, but instead found increased Mg, Cu, and Zn concentrations under Mn toxicity (Fig. 2). An increase in Zn was also observed in the high Mn accumulation and Mn toxicity-sensitive rice mutants *osmtp8.1*^16^. Under oxidative stress conditions, Zn uptake is enhanced and correlated with enhanced SOD activity^93^. Similar to oxidative stress conditions, excess Mn concentrations also upregulate SOD activities^38,45^. Among the different metal types of SOD, Gonzálets et al.^26^ reported that Cu/Zn-SOD and Mn-SOD activities were significantly increased under excess Mn conditions. Furthermore, the Mg supply rescues Mn toxicity symptoms^3^. From these observations, we suggest that the requirement for these minerals might be upregulated under Mn-toxic conditions.

Our results suggest that chloroplasts contain homeostatic systems that evade excessive Mn accumulation. In vitro studies have shown that excess free Mn stimulates ROS production by PSII and causes severe oxidative damage to thylakoid lipids and PSII^94^. Although the Mn concentration was high (Fig. 2), no clear evidence of severe damage to PSII was detected (Fig. 3a,g,h,i). Moreover, clear evidence of Mn^2+^-binding to Rubisco was also not observed (Supplementary Figs. S1 and S2). These results suggest that chloroplasts function as protective features to avoid Mn hyperaccumulation. Based on previous studies, two mechanisms can be considered. The first mechanism involves binding Mn to the thylakoid membranes. Although involves modulation of the membrane structure of the thylakoid membranes, no inhibitory effect on photosynthetic activity was observed^95,96^. The binding of Mn to the thylakoid membrane could be a storage system for Mn homeostasis. The second mechanism is sequestration or limiting Mn transport within the chloroplasts. Indeed, Mn distributed to the chloroplasts decreased under Mn-toxic conditions in rice^39^.

The growth defect due to symplastic Mn toxicity could be caused by indirect inhibition of carbon catabolism due to the lower carbohydrate concentration in the leaves. A decrease in respiration rate under Mn toxicity has been reported in wheat and cotton plants^29,97^. We further observed a decrease in leaf respiration, but not under sucrose feeding conditions (Fig. 5a,c). Moreover, no decreases in mitochondrial enzyme activities, including the TCA cycle and respiratory electron transport chain enzymes, were observed, except for the NAD-malic enzyme (Fig. 5). The physiological importance of the NAD-malic enzyme is highly limited in C3 plants, and a pronounced phenotype has not been observed in the mutants in *Arabidopsis*^98^. This could be because of the lower contribution to the supply of pyruvate into the TCA cycle by NAD-malic enzyme activity^99^. These observations suggest that the significant decrease in NAD-malic enzyme activity could be independent of the growth defect and decreased respiration rate under Mn-toxic conditions (Figs. 1 and 5). However, the change in amino acid composition could be influenced by a decrease in the NAD-malic enzyme^98^. From these observations, the decreased respiration rate was caused by a decrease in carbohydrate concentrations due to the suppression of the CO_2_ assimilation reaction (Fig. 4). Because carbohydrate catabolism during the night is a determinant of plant growth^51^, a decrease in the capacity of carbon catabolism would cause growth defects in rice under Mn toxicity.

Interestingly, Hibberd and Quick^100^ reported that similar to C4 plants, the NAD-malic enzyme activity is high in the bundle-sheath cells of C3 plants. Here, we found that the bundle-sheath cells turned brown under Mn-toxic conditions (Fig. 7). Based on a previous study on apoplastic Mn toxicity, brown spots contained oxidised phenol, oxidised Mn, and callose. Fernando et al.^18^ showed that wheat leaves concentrate Mn in the bundle-sheath cell; however, this specific localisation was not observed in soybean and canola, suggesting that the bundle sheath cell could function as storage organs for attenuating Mn toxicity in monocots. The Mn accumulation in the bundle sheath cell may suppress NAD-malic enzyme activity in leaves. These results provide an insight into the function of bundle-sheath cells in C3 monocots.

## Conclusion

**Figure 9:**
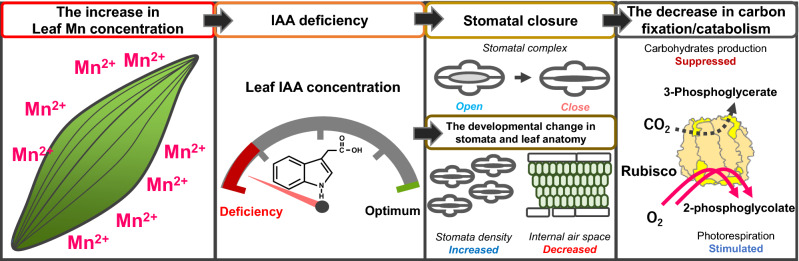
Visual scheme of symplastic Mn toxicity to suppress CO_2_ assimilation. When Mn is excessively accumulated in leaves, the leaf IAA concentration is lowered, which might stimulate IAA degradation activities under Mn toxicity^57^. As a short-term effect, the decrease in IAA concentration affects stomatal opening^73^. Subsequently, as a long-term effect, the auxin-dependent signal transduction involving ARF transcriptional factor is perturbated in the leaves to cause an IAA-deficient phenotype, changing both the stomatal and leaf anatomical structures^71,72^. The change in stomatal function and leaf structure severely limits CO_2_ diffusion to the chloroplasts, and CO_2_ assimilation by Rubisco is inhibited. In contrast, the photorespiration reaction limits the photosynthetic electron activities to cause ROS production^82^. The decreased sugar production efficiency in photosynthesis suppresses sugar catabolism. Therefore, growth is inhibited under Mn-toxic conditions.

We proposed that the disturbance of IAA homeostasis is an initial cause of the inhibition of CO_2_ assimilation and photoinhibition caused chlorosis in the leaves (Fig. 9). As a short-term effect, a decrease in IAA suppresses stomatal opening. Subsequently, the decrease in IAA concentration modifies leaf anatomy and stomatal development in newly emerged leaves as a long-term effect. The decreased CO_2_ assimilation rate and increased photorespiration rate decelerate carbon acquisition for catabolism; thus, vegetative growth is suppressed. To our knowledge, very few studies have previously focused on the relationship between auxins and Mn toxicity^57^. In this study we did not separately quantify the Mn concentration between the apoplast and symplast. Based on our discussion, the emergence of symplastic Mn toxic symptoms could be related to the emergence of apoplastic Mn toxicity because the apoplast is an important site for IAA transport. In future research, the relationship between apoplastic and symplastic Mn toxicity should also be investigated. The present study would develop a new strategy to avoid Mn toxicity.

## Materials and methods

### The use of plant materials and ethical approval statement

Rice seeds (*Oryza sativa* L. cv. Nipponbare) that we used in the present experiments were transferred from the Institute of Crop Science in the National Agriculture and Food Research Organization (Tsukuba, Ibaraki, Japan) to Tohoku University (Sendai, Miyagi, Japan) and Setsunan University (Hirakata, Osaka, Japan) with a material transfer agreement. All the experiments were conducted in compliance with the guideline and regulations of this contract. We also followed the relevant guidelines and regulations of the government of Japan, the Tohoku University, and Setsunan University when proceeding with all the experiments in the present study and writing this manuscript.

### Plant materials and plant growth conditions

Rice plants were grown in hydroponic culture as reported in a previous study^101^. Seeds were imbibed at 30 °C for 3 days and subsequently grown on a plastic net floating on tap water for 3 weeks. After the emergence of the third leaf, each rice plant was cultivated separately in a 1.5 L plastic pot containing a hydroponic solution of two different Mn concentrations. We employed two Mn concentration conditions by changing the MnSO_4_ application to 0.5 µM (control conditions) and 200 µM (Mn-toxic conditions)^27^, based on the nutrient composition of the hydroponic solution in the previous study^101^. The pH of the hydroponic culture was adjusted to 5.2 using HCl, and the solution was replenished twice a week. The chamber was maintained at 60% relative humidity with a 14 h light (28 °C) and 10 h dark (25 °C) photoperiod. The light intensity was 500–600 µmol photons m^−2^ s^−1^. All physiological, structural, and genetic analyses were performed for fully and newly expanded leaves after 70 days of germination, and the analyses were completed before heading. For each experiment, biologically independent rice plants grown in independent hydroponic pots were used, and the physiological, biochemical, and morphological experiments were conducted separately using rice plants grown under control and Mn-toxic conditions.

### Leaf photosynthesis and respiration measurements in rice plants

Gas exchange analysis, chorophyll (Chl) fluorescence, and oxidised reaction centre Chl in PSI (P700^+^) were simultaneously measured using a combined system of Li-6400 (Li-COR Inc., Lincoln, USA), Mini-PAM, and PAM-101 equipped with a dual-wavelength emitter-detector unit (ED-P700DW) (Heinz Walz GmbH, Effeltrich, Germany) with a cold halogen lamp. Ambient air (40 Pa CO_2_ and 21 kPa O_2_) and pure CO_2_ gas were mixed to maintain the CO_2_ concentration during the measurements. The gases were saturated with water vapour at 18.0 ± 0.1 °C, and the leaf temperature was maintained at 28 °C. The Chl fluorescence parameters Y(II), Y(NPQ), and Y(NO) were calculated as described by Baker^102^, and a measuring light (0.1 µmol photons m^–2^ s^–1^) and a saturated pulse (10,000 µmol photons m^–2^ s^–1^, 600 ms) were employed to determine Fo (minimum fluorescence yield), Fm (maximum fluorescence yield), Fm’ (maximum fluorescence yield under the illumination), and Fs (steady-state fluorescence yield) to calculate the photosynthetic parameters of PSII. The oxidation–reduction state of P700 in PSI was determined according to the method described by Klughammer and Schreiber^103^. The maximum oxidation level of P700 (Pm) was obtained using a saturated pulse under far-red light illumination, and the oxidation–reduction state of P700 was determined using the oxidation level of P700 at the steady-state (P) and the maximum oxidation level of P700 under illumination (Pm′). PSI photosynthetic parameters [Y(I), Y(ND), and Y(NA)] were calculated using these oxidation–reduction states of P700, as determined by the saturated pulse method.

The CO_2_ emission rate was measured using Li-6400 in the gaseous phase. The O_2_ absorption rate was measured using an O_2_-electrode (CB1D; Hansatech Instruments Ltd., King’s Lynn, UK) in the aqueous phase at 25 °C^54^. For efficient absorption of the chemical reagent into leaves in the aqueous phase, the leaf surface was washed with 10% (v/v) dimethyl sulfoxide, and the leaves were finely cut up. The O_2_ absorption rate was measured in the reaction buffer [50 mM N-(2-hydroxyethyl)piperazine-*N*'-2-ethanesulfonic acid (HEPES)-KOH (pH 6.6), 10 mM 2-(*N*-morpholino)ethanesulfonic acid, 0.2 mM CaCl_2_, 50 mM sucrose]. To evaluate the uncoupled respiration rate and alternative oxidase (AOX) and cytochrome *c* oxidase (COX) activities in the mitochondria, 10 µM carbonyl cyanide m-chlorophenyl hydrazine, 2 mM n-propyl gallate, and 2 mM KCN were added sequentially.

### Quantification of leaf mineral elements, chlorophyll and P700 content

Leaf mineral elements were analysed according to the method of Takagi et al.^104^. Briefly, the leaf blades were dried at 70 oC and ground using a homogeniser. To determine the mineral content, except for that of N, the dry matter was digested overnight in an acid mixture (HNO_3_:H_2_SO_4_:HClO_4_ = 5:1:2). Subsequently, the solution was heated at 150 °C for 30 min on a heat block. After cooling, the solution was heated again at 200 °C for 1 h. After decomposition, the mineral contents were quantified by inductively coupled plasma optical emission spectroscopy (ICP-OES) (iCAP™ 7200; Thermo Fisher Scientific Inc., Waltham, USA). To measure the total N concentration in the leaves, dried leaves were decomposed using 60% (v/v) H_2_SO_4_ with 30% (v/v) H_2_O_2_. The N concentration was determined using Nessler’s reagent in a decomposed solution after the addition of 10% (w/v) potassium sodium tartrate and 2.5 M NaOH, and the absorbance was measured at 420 nm^81^.

The leaf Chl content was quantified using fresh samples, as described previously^104^. In brief, leaf segments were incubated in *N, N*-dimethylformamide at 4 °C overnight, and the absorbance values of the aliquots were measured at 750 nm, 663.8 nm, and 646.8 nm to calculate the chlorophyll content.

The reaction centre chlorophyll (P700) was quantified in leaves using a Dual-PAM 100 (Heinz Walz GmbH, Effeltrich, Germany). The absorbance changes at 830 nm referenced by that at 875 nm were calculated by illuminating the FR light and a saturated pulse^103,105^.

### Carbohydrates quantification in leaves

Leaves were sampled at the end of the day (1 h before the start of the dark period) and at the end of the night (1 h before the start of the light period); subsequently, leaves were frozen using liquid nitrogen. Glucose, sucrose, and starch were extracted from leaves according to the protocol of a previous study^104^ and quantified using the Enzytech™ D-Glucose/Sucrose kit (R-Biopharm AG, Darmstadt, Germany).

### Quantification of leaf amino acid contents

The leaves were homogenised using 10 mM HCl in liquid nitrogen. After centrifugation (15,000×*g*, 4 °C, 5 min), the supernatant was applied to a centrifugation filter (Amicon Ultra 0.5 mL 3 K device; Merck KGaA, Darmstadt, Germany), and the fraction containing free amino acids was obtained by centrifugation (15,000×*g*, 4 °C, 10 min). The amino acids were labelled using the AccQ-Tag Ultra Derivatization Kit (Waters Co., Milford, USA) according to the manufacturer’s instructions, and the free amino acids were separated using an HPLC system. The HPLC system consisted of a 305 piston pump as the system controller and a 306 pump as a multi-pump application equipped with an 811D dynamic mixer and 805 manometric module, using the Trilution LC software (Gilson, Villiers le Bel, France). The sample solution was manually injected into an HPLC system. The derivative amino acids were separated on a Sepax Bio-C18 (φ4.6 × 250 mm) (Sepax Technologies, Inc., Newark, USA) and eluted with 100% solvent A [5% (v/v) methanol in 20 mM CH_3_COONa (pH 6.5)] for 4 min after injection and by a linear gradient of 94% solvent A to 30% solvent B (100% acetonitrile) for another 65 min. The column was washed with 100% solvent B for 20 min and equilibrated with 100% solvent A for 16 min. The flow rate was 0.6 mL min^−1^, and the column temperature was maintained at 25 °C by incubation in a hot pocket (Thermo Fisher Scientific Inc., Waltham, USA). The peaks derived from derivatized amino acids were detected using a UV/VIS-155 (Gilson, Villiers le Bel, France).

### TCA cycle enzyme activities

The TCA cycle enzyme activities in the leaf blades were determined according to Noguchi and Terashima^106^. Briefly, the leaves were homogenised in extraction buffer [100 mM HEPES–KOH (pH 7.5), 10 mM KH_2_PO_4_, 0.5 mM EDTA-Na, 10 mM dithiothreitol, 0.05% (v/v) TritonX-100, and 20% (v/v) glycerol] with polyvinylpyrrolidone. The reaction medium was prepared for each enzyme according to the protocol of a previous study, and the enzyme activity was determined at 30 °C using a UV-160A equipped with a temperature control system (Shimadzu, Kyoto, Japan).

### Structural analysis of stomata complex and leaf cross-section

For scanning electron microscopic analysis, the leaves were fixed in half-strength Karnovsky’s solution [50 mM phosphate buffer (pH 7.2), 2.5% (v/v) glutaraldehyde, 2% (v/v) paraformaldehyde] with vacuum infiltration for 3 h. After fixation, the leaves were washed with phosphate buffer and dehydrated using a dilution series of ethanol, followed by t-butanol. Leaves were dried using a t-butanol freeze drier (Vacuum Device Inc., Ibaraki, Japan) and coated with Pt (JEC-300FC; JEOL Ltd., Tokyo, Japan). The stomatal complex structure was monitored using a JSM-IT200 scanning electron microscope (SEM) (JEOL Ltd., Tokyo, Japan). The size of the stomatal complex, comprising the paired guard cells, the paired subsidiary cells, and the pore itself, and the stomatal density were analysed using SEM Operation software (JEOL Ltd., Tokyo, Japan). Under each growth condition, the size of the stomata complex and the stomatal density were analysed on an independent section of the leaves as indicated in figure legends of six biologically independent plants.

To analyse the vertical and horizontal leaf sections, leaves were sectioned and fixed with formalin-acetate-alcohol (FAA) [63% (v/v) ethanol, 5% (v/v) acetic acid, 5% (v/v) formalin] with vacuum infiltration for 1 h, followed by incubation at 4 °C for 2 days. The leaves were dehydrated using a dilution series of ethanol, followed by n-butanol. The dehydrated leaves were embedded in paraffin and sectioned at 10 µm thickness using an RX-860 microtome (Yamato Kohki Industrial Co., Ltd., Saitama. Japan). The FAA-fixed leaf sections were stained with 0.05% (w/v) toluidine blue O. Subsequently paraffin was removed using xylene solution. To investigate the intact leaf sections, leaves were embedded in 50 mM phosphate buffer (pH 7.2) containing 5% (w/v) agar. The agar-embedded leaves were sectioned at a thickness of 30 µm using a DTL-1000 vibratome (Dosaka EM Co., Ltd., Kyoto, Japan). Intact leaf sections and FAA-fixed leaf sections were analysed using an optical microscope (BX53; Olympus Co., Japan).

### Indole acetic acid quantification

The IAA content in leaves was quantified using a gas chromatography and mass spectrometry (GC–MS) system, according to Nghi et al*.*^107^. Briefly, the leaves were homogenised in 70% (v/v) cold acetone. After centrifugation, the supernatant was reduced to the aqueous phase by vacuum centrifugation equipped with a cold trap. Subsequently, HCl (0.1 mM) was added to adjust the pH to 2.8, and diethyl ether was added to dissolve the IAA into the diethyl ether fraction. The diethyl ether fraction was dried under vacuum and subsequently dissolved in *N*,*O*-bis(trimethylsilyl) tri-fluoroacetamide (BSFTA) containing 1% trimethylchlorosilane. IAA was silylated at 70 °C for 1 h. The silylated IAA was identified using a GC–MS-QP2010 SE (Shimadzu, Kyoto, Japan) with a DB-WAX column. The conditions for GC–MS analysis were the same as described previously^108^. The silylated IAA was identified by fragment ion 202 (*m*/*z*) and molecular ion 319 (*m*/*z*)^107,109,110^.

### Gene expression analysis

To investigate gene expression in rice leaves, mRNA was isolated from the leaf blades according to Suzuki et al.^111^. After mRNA isolation, cDNA was synthesised using PrimeScript™ RT Reagent Kit (Takara Bio Inc., Shiga, Japan), and the mRNA content was quantified by real-time PCR using the LightCycler® 96 System (Roche Diagnostics K.K., Tokyo, Japan) with a KAPA SYBR FAST One-Step qRT-PCR Kit (Nippon Genetics Co., Ltd, Tokyo, Japan). The primer sequences used for real-time PCR analyses are listed in Supplementary Table S1.

### Simulation of CO_2_ fixation rate according to biochemical photosynthetic model

The Rubisco limited CO_2_ fixation rate was calculated from the equation of von Caemmerer and Farquhar^48^:

1 $$ {\text{A}} = {\text{EV}}_{{\text{c}}} \left( {{\text{Cc }} -  \Gamma *} \right){/}\left[ {{\text{Cc }} + {\text{ K}}_{{\text{c}}} \left( {{1 } + {\text{O}}{/}{\text{K}}_{{\text{o}}} } \right)} \right] - {\text{ Rd}}, $$

where E is the amount of Rubisco protein (assumed to be 5.3 μmol m^−2^, from rice grown with hydroponic solution as in the study described by Suganami et al.^49^, V_c_ is the Rubisco activity of carboxylation, Cc is the chloroplastic CO_2_ partial pressure, O is the partial pressure of O_2_ in the chloroplast (assumed to be the same as in the atmosphere, 21 kPa), K_c_ and K_o_ are the Michaelis–Menten constants for CO_2_ and O_2_, and Rd is the day respiration (assumed to be 1.0 μmol m^−2^ s^−1^). Γ* is the CO_2_ compensation point of photosynthesis in the absence of Rd ,which is defined as follows:

2 $$ \Gamma * = 0.{\text{5V}}_{{\text{o}}} {\text{K}}_{{\text{c}}} {\text{O}}{/}{\text{V}}_{{\text{c}}} {\text{K}}_{{\text{o}}} , $$

where V_o_ is Rubisco activity of oxygenation. Mg^2^^+^-binding Rubisco kinetics were taken from Makino et al.^50^. Mn^2+^-binding Rubisco kinetics were calculated by multiplying the average ratio of Mn^2+^/Mg^2+^-binding Rubisco kinetics and Mg^2+^-binding Rubisco kinetics. The average ratio of Mg^2+^/Mn^2+^-binding Rubisco kinetics was calculated using data from Bloom & Kameritsch^47^. The Rubisco kinetics are summarised in Supplementary Table S2. The photorespiration rate were was calculated from the equation of von Caemmerer & Farquhar^48^:

3 $$ {\text{F}} = 0.{\text{5EV}}_{{\text{o}}} {\text{O}}{/}({\text{O}} + {\text{ K}}_{{\text{o}}} \left( {{1 } + {\text{Cc}}{/}{\text{ K}}_{{\text{c}}} } \right), $$

### Statistical analysis

All measurement data are expressed as means ± standard deviation (SD), or box plots with bars indicating the 1.5 × interquartile range (IQR) and with squares indicating the mean value of at least three independent biological analyses. Significant differences in physiological and biological parameters between the control- and Mn-toxic conditions were detected using non-parametric Kruskal–Wallis test. All statistical analyses were performed using Origin Pro 2020 (LightStone Corp., Tokyo, Japan).

## Supplementary Information

Supplementary Information.

## Data Availability

The data used or analysed during this study are available from the corresponding author upon reasonable request.
