# Supplementary material for: Manganese toxicity disrupts indole acetic acid homeostasis and suppresses the CO2 assimilation reaction in rice leaves
Source: Sci Rep. 2021 Oct 22;11:20922. doi: 10.1038/s41598-021-00370-y (PMC8536708; doi:10.1038/s41598-021-00370-y)
Supplement: Supplementary file 1 — Supplementary Information. [file 41598_2021_370_MOESM1_ESM.pdf]

TITLE: Manganese toxicity disrupts indole acetic acid homeostasis and suppresses the CO_2 assimilation reaction in rice leaves

RUNNING TITLE: *Mechanism of symplastic Mn toxicity in leaves*

AUTHORS: Daisuke Takagi[1,2]*, Keiki Ishiyama[2], Mao Suganami[2 ‡], Tomokazu Ushijima[1], Takeshi Fujii[1], Youshi Tazoe[2, †], Michio Kawasaki[1], Ko Noguchi[3], Amane Makino[2]

AUTHORS AFFILIATIONS:

[1] Faculty of Agriculture, Setsunan University, Hirakata, Osaka 573-0101, Japan

[2] Graduate School of Agricultural Science, Tohoku University, Sendai, Miyagi 980-8572, Japan

[3] Department of Applied Life Science, School of Life Sciences, Tokyo University of Pharmacy and Life Sciences, Hachioji, Tokyo, 192-0392 Japan

[†] Present address; Faculty of Agro-Food Science, Niigata Agro-Food University, Tainai, Niigata, 959-2702 Japan

[‡] Present address; Faculty of Food and Agricultural Sciences, Fukushima University, Kanayagawa, Fukushima, 960-1296, Japan

***For correspondence**

Corresponding author: Daisuke Takagi

E-mail: daisuke.takagi@setsunan.ac.jp

TEL/FAX: +81-72-896-5395

SUPPLEMENTARY INFORMATION

Figure S1.; The simulated CO_2 assimilation in leaves containing Mg-binding Rubisco and Mn-binding Rubisco.

Figure S2.; The relationship between g_s and CO_2 fixation rate under the control and the Mn-toxic conditions.

Figure S3.; The sucrose-feeding respiration activities and mitochondrial respiratory electron transport activities depending on ATP synthase (uncoupled), COX, and AOX based on leaf fresh weight.

Figure S4.; The enzyme activities involved in the TCA cycle calculated on the fresh weight basis.

Figure S5.; PSI content in leaves grown under the control and Mn-toxic conditions

Table S1.; The primer list for the gene expression analysis in rice leaf blade

Table S2.; Mg^{2+}-binding Rubisco and Mn^{2+}-binding Rubisco kinetics.

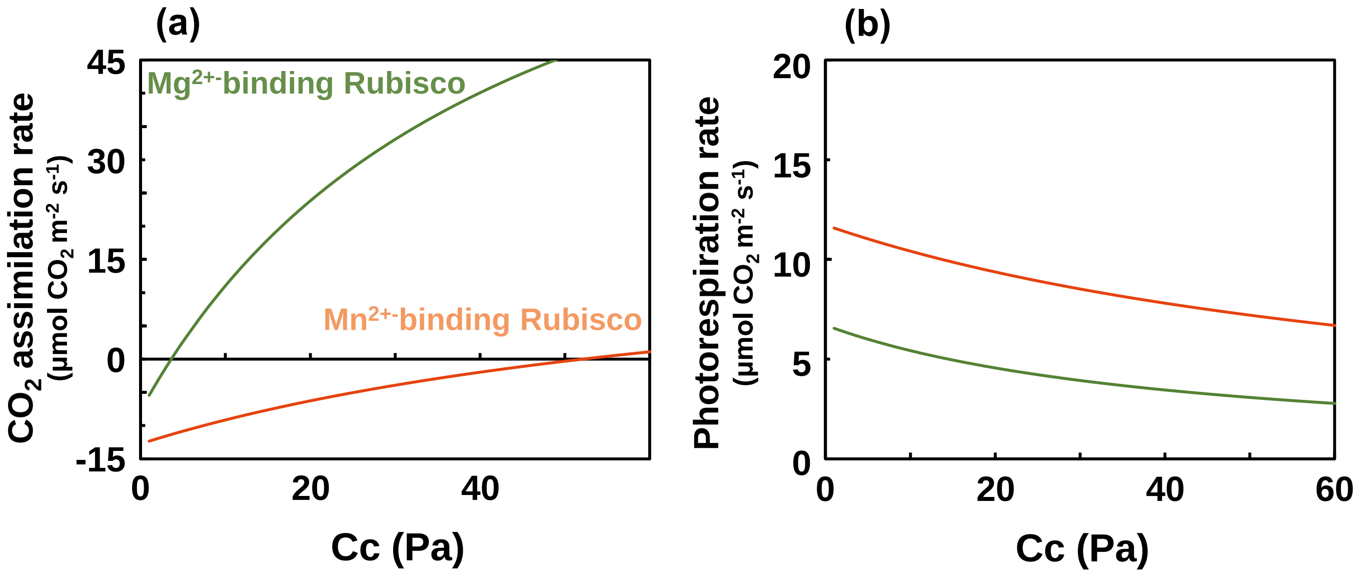

Figure S1.

The simulated Rubisco-limited CO_2 assimilation rate (a) and photorespiration rate (b) in leaves containing Mg^{2+} binding Rubisco and Mn^{2+}-binding Rubisco. Rubisco-limited CO_2 assimilation rate were calculated from Equation (1). We assumed that Rubisco content was the same as in our previous study[49]. Mg^{2+}/Mn^{2+} binding Rubisco kinetics were summarized in Table. S2. Photorespiration rate were calculated from Equation (3). The green line indicates the simulation using Mg^{2+}-binding Rubisco kinetics and the pink line indicates the simulation using Mn^{2+}-binding Rubisco kinetics.

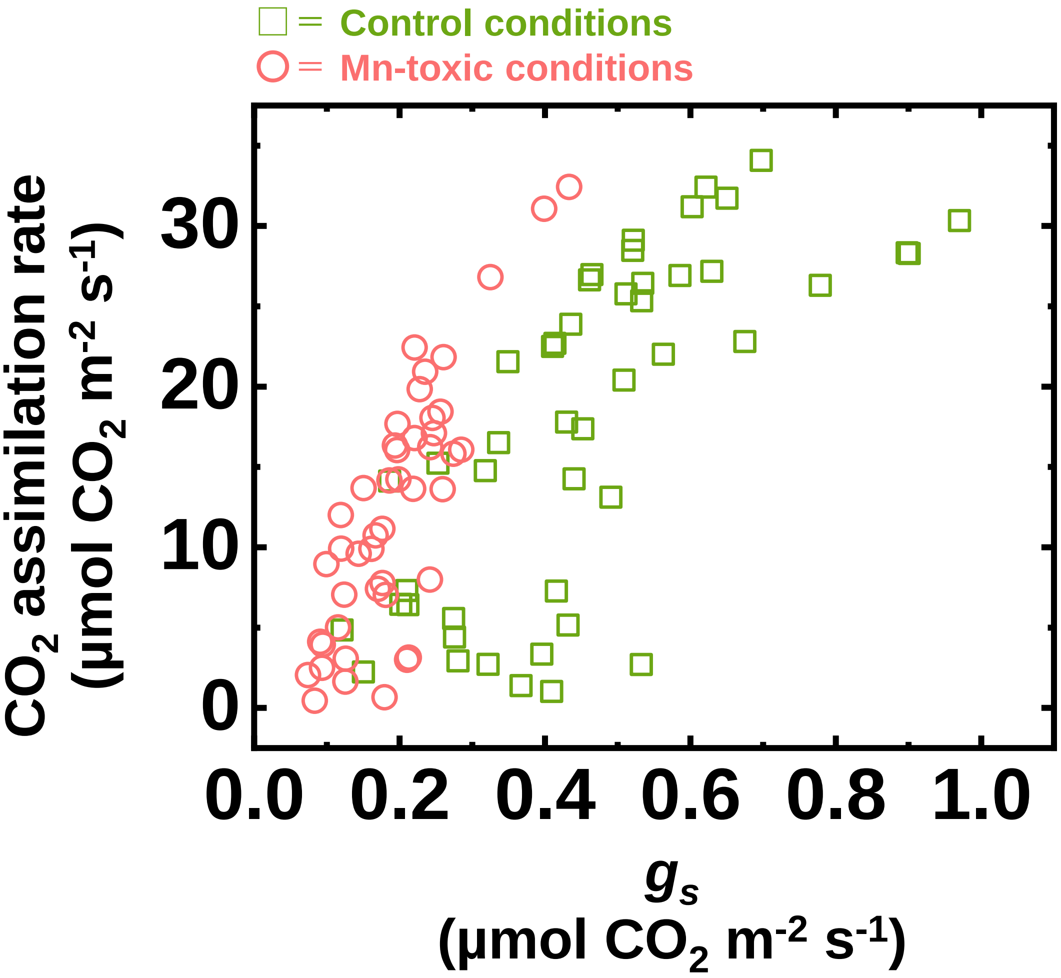

Figure S2.

The relationship between g_s and CO_2 assimilation rate under the control and the Mn-toxic conditions. The data shown here is identical to the results shown in Figure 3. The green squares indicate the result of the control conditions, and the pink circles indicate the result of the Mn-toxic conditions.

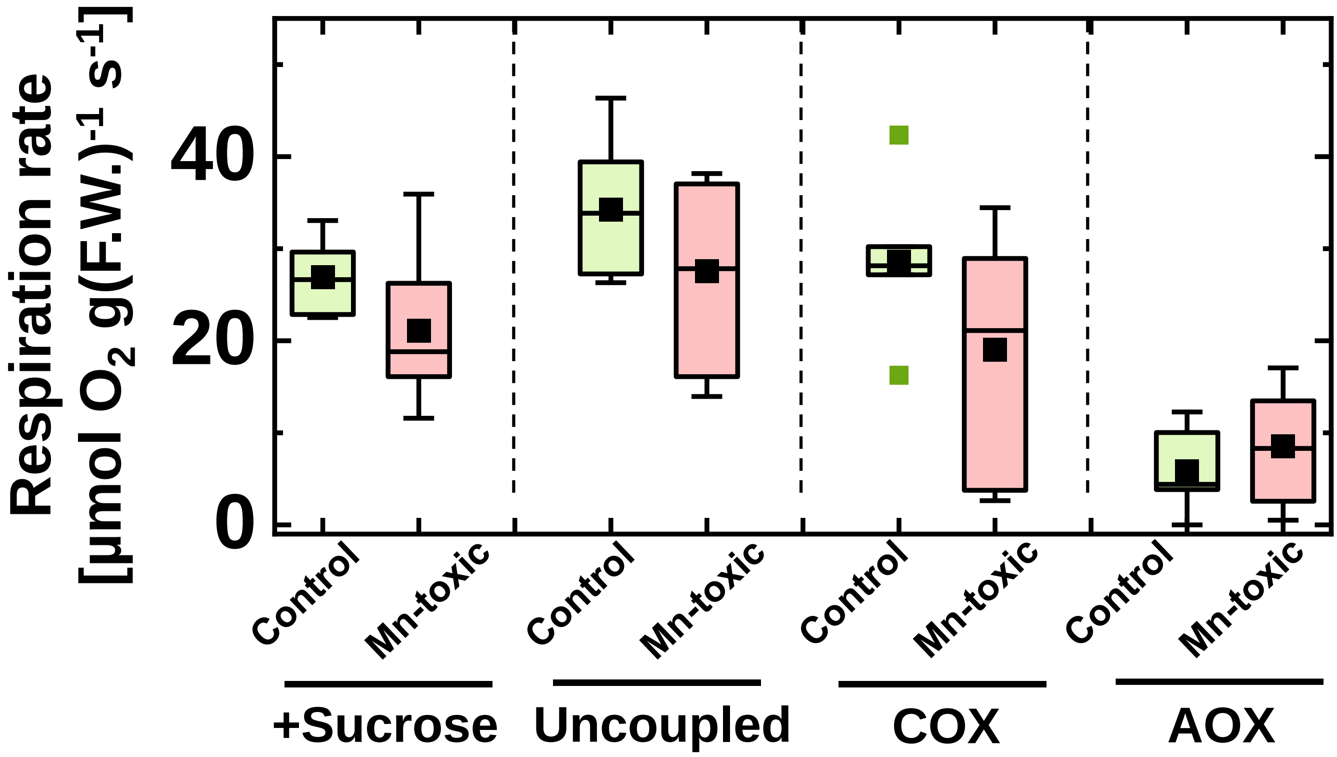

Figure S3.

The sucrose-feeding respiration activities and mitochondrial respiratory electron transport activities depending on ATP synthase (uncoupled), COX, and AOX based on leaf fresh weight (n = 7). Data are shown as box plots obtained from independent biological replicates as indicated, and black squares indicate the mean value, and bars indicate the 1.5 × IQR (interquartile range) of the data. The green boxes indicate the results of the control-conditions, and the red boxes indicate those of the Mn-toxic conditions.

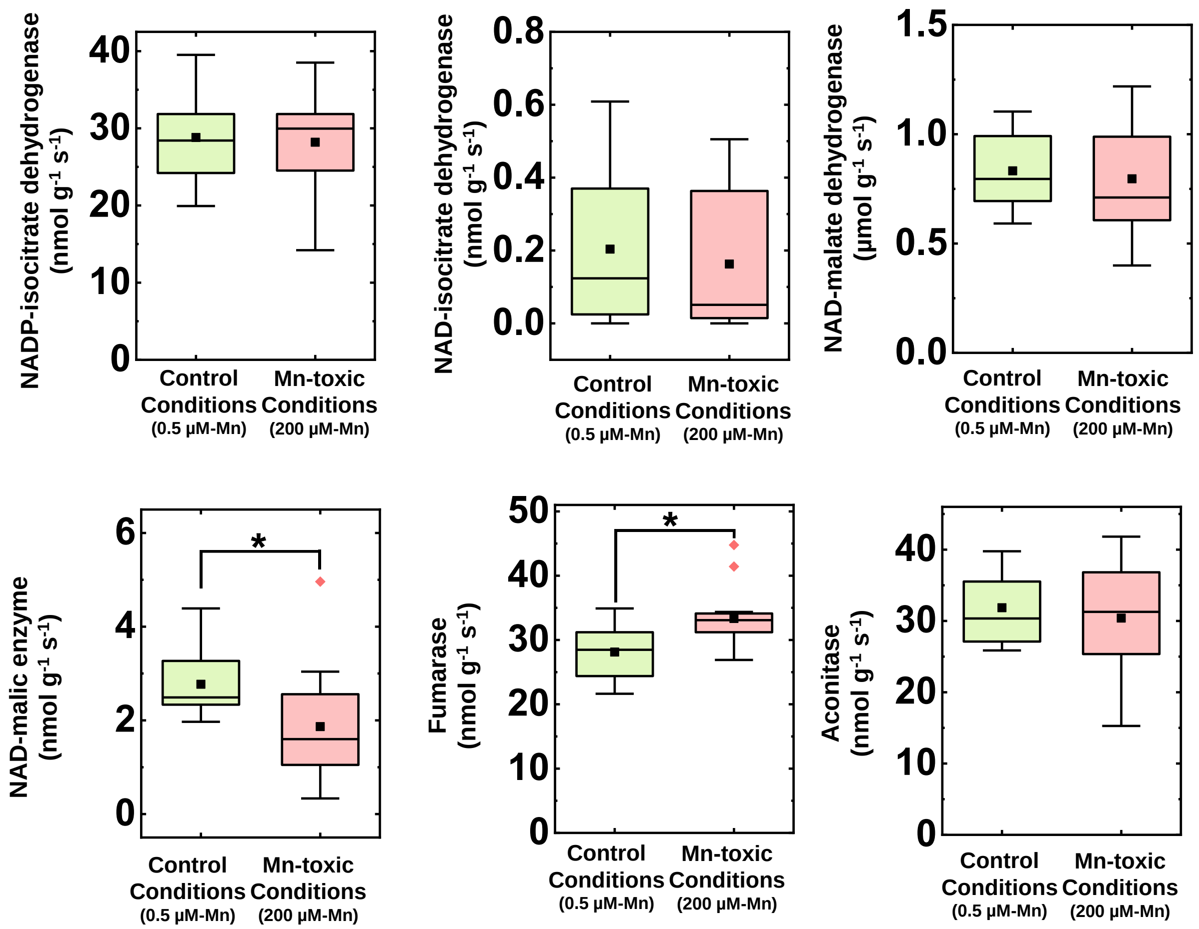

Figure S4.

The enzyme activities involved in the TCA cycle, respectively (n = 13-14). The activities were expressed on a leaf-fresh weight basis. Data are shown as box plots, and black squares indicate the mean value, and bars indicate the 1.5 × IQR (interquartile range) of the data obtained from independent biological replicates as indicated. The green boxes indicate the results of the control-conditions, and the red boxes indicate those of the Mn-toxic conditions. Asterisks showed significant differences between the condition- and the Mn-toxic conditions (*; $p < 0.05$, **; $p < 0.01$, Kruskal-Wallis test).

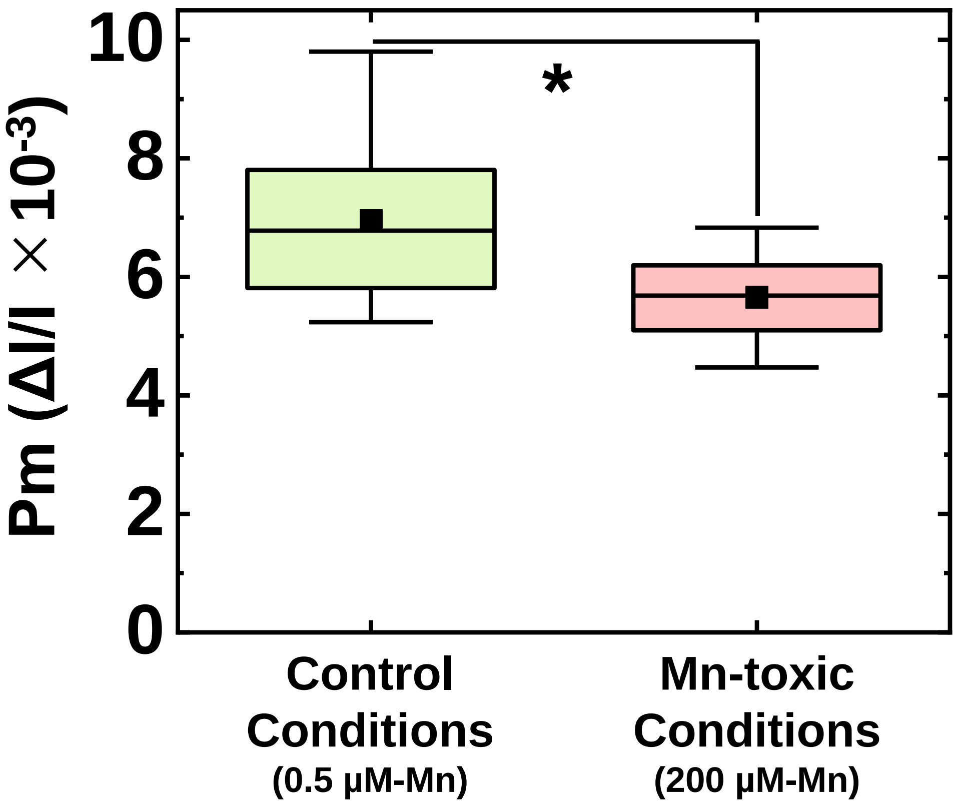

Figure S5.

PSI content in leaves grown under the control and Mn-toxic conditions (n = 10). The PSI content was determined by the absorbance change by P700 reaction center chlorophyll in leaf area basis (1.0 cm^2). Data are shown as box plots, and black squares indicate the mean value, and bars indicate the 1.5 × IQR (interquartile range) of the data obtained from independent biological replicates as indicated. The green boxes indicate the results of the control-conditions, and the red boxes indicate those of the Mn-toxic conditions. Asterisks showed significant differences between the condition- and the Mn-toxic conditions (*; $p < 0.05$, Kruskal-Wallis test).

Supplemental Table S1

The primer list for the gene expression analysis in rice leaf blade

**Gene name**	**Gene ID**		Primer sequence (5' to 3')
OsARF1	Os11g0523800	Left	ACTGGATATGAGCCGTCAGC
		Right	GGTGTCTTCGTGGTTGACCT
OsARF15	Os05g0563400	Left	TCCCTCCACTGGATTACAGC
		Right	CAAATGCACTCCAACCTGTG
OsARF16	Os01g0236300	Left	TGACCCGGATCAAGAGAATC
		Right	GGATCTTGCAGAAGGAGTGC
OsAUX/IAA1	Os01g0178500	Left	CGCTCCAGGACAAGTTCTTC
		Right	GTACTCCGTCCCGTTCACC
OsAUX/IAA24	Os07g0182400	Left	AAGGCACAGGTGGTAGGATG
		Right	ATCACCACCCTTCTTGGTTG
OsDAO	Os04g0475600	Left	GAGAGGATGCACTCGCTGAT
		Right	ACGGAGTCCTGCGTGTAGTT
OsACT1-1	Os03g0718100	Left	ATAGCATGGGGGAGAGCATA
		Right	CGTCTGCGATAATGGAACTG
OsACT1-2	Os03g0718100	Left	TCCATCTTGGCATCTCTCAG
		Right	GTACCCGCATCAGGCATCTG

Table S2 Mg^{2+}-binding Rubisco and Mn^{2+}-binding Rubisco kinetics.

	Mg^{2+}-binding Rubisco	Mg^{2+}-binding Rubisco
**Vc (mol mol^{-1} Rubisco s^{-1})**	17.3	3.9
**Vo (mol mol^{-1} Rubisco s^{-1})**	5.7	5.3
**Kc (Pa)**	23.6	13.4
**Ko (kPa)**	26.2	4.3

Mg^{2+}-binding Rubisco kinetics were taken from Makino et al.[50]. Mn^{2+}-binding Rubisco kinetics were calculated by multiplying the average ratio of Mn^{2+}/Mg^{2+}-binding Rubisco kinetics and Mg^{2+}-binding Rubisco kinetics. The average ratio of Mg^{2+}/Mn^{2+}-binding Rubisco kinetics was taken from Bloom & Kameritsch[47].
